# Adapting the self-assessment of contextual fit scale for implementation of evidence-based practices in adolescent HIV settings

**DOI:** 10.1186/s43058-022-00349-4

**Published:** 2022-10-22

**Authors:** Karin Coyle, April Idalski Carcone, Seyram Butame, Meardith Pooler-Burgess, Jason Chang, Sylvie Naar

**Affiliations:** 1 ETR, 5619 Scotts Valley Dr., Suite 140, Scotts Valley, CA 95066 USA; 2 Division of Behavioral Sciences, Family Medicine and Public Health Sciences, Wayne State University, Integrated Biosciences Building (IBio) #3128, 6135 Woodward, Detroit, MI 48202 USA; 3 Center for Translational Behavioral Science, College of Medicine, Florida State University, 2010 Levy Ave., Bldg. B, Suite 0266, Tallahassee, FL 32310 USA

**Keywords:** Implementation science, Contextual fit measurement, Adolescent, Evidence-based practices

## Abstract

**Background:**

Contextual fit is an important variable in the implementation of evidence-based programs (EBPs). The objectives of the current study were to examine the psychometric properties of the adapted Self-Assessment of Contextual Fit (SACF) measure for HIV clinical care settings (calling it SACF-HIV) and explore how perceptions of contextual fit varied across two different interventions (an intervention to scale up tailored motivational interviewing and an individually focused HIV prevention intervention) and 12 clinical sites.

**Methods:**

We collected SACF-HIV data as part of a larger cross-project implementation science study (ATN 153). The study sample includes 128 clinicians, community health workers, interventionists, adherence counselors, and other members of the prevention and care team who engage in the implementation of EBPs at 12 HIV prevention and clinical care sites in the USA. We assessed the internal consistency of the SACF-HIV using Cronbach’s alpha and examined the sub-dimensionality of the scale with an exploratory factor analysis. To explore concurrent validity, we examined Pearson’s correlation coefficients between the adapted scale and fit-related sub-scale scores from the Evidence-Based Practice Attitudes Scale-50 (EBPAS-50). Variation in perceptions of fit by intervention was examined using descriptive statistics.

**Results:**

Internal consistency of the adapted scale was strong (*α*=0.895). Factor analyses revealed two sub-scales—one capturing general insights regarding contextual fit, such as perceptions of skill, experience, and alignment with client needs (loadings ranging from .5 to .84), and a second centering perceptions regarding implementation support, such as resources and administrative support (loadings ranging from .89 to .97). Concurrent validity was supported by statistically significant correlations in the expected direction with EBPAS-50 fit-related sub-scales (*r*=.33–.35, *p* ≤ 0.05). SACF-HIV mean fit scores varied by intervention and the difference was statistically significant (2.78 vs. 2.53, *p* < 0.05).

**Conclusions:**

There are relatively few tools assessing perceptions of contextual fit in HIV clinical settings. These results suggest the 12-item adapted SACF is a reliable, valid global assessment of perceptions of contextual fit and implementation support. The SACF-HIV can be used by practitioners and researchers interested in understanding an implementation context when planning to prepare and support EBP implementation.

**Trial registration:**

TMI ClinicalTrials.gov NCT03681912; YMPH ClinicalTrials.gov NCT03488914

Contributions to the literature

- Adds to the growing body of implementation science research in HIV clinical settings
- Adapts a brief measure of perceived contextual fit specifically for HIV clinical settings preparing for the implementation of evidence-based practices
- Provides reliability and validity data supporting a brief assessment tool for examining HIV clinical implementation contexts

## Background

Despite a fast-growing implementation science literature, the implementation of EBPs into HIV settings, particularly youth-serving HIV clinics, has received less attention [1]. Successful integration of evidence-based HIV prevention and care strategies is one of several pillars of the US strategy for *Ending the HIV Epidemic* [2], underscoring the importance of continued implementation science research and a focus on factors that facilitate implementation. Funders, such as the National Institutes of Health, have highlighted implementation science research as a priority within HIV/AIDS (e.g., through their HIV Prevention and Treatment Implementation Science Program and through updated HIV/AIDS research priorities).

Contextual fit, or the match between intervention elements, strategies, and procedures with the values, needs, skills, and resources of those using the intervention [3], is considered an important variable in the implementation of evidence-based programs (EBPs) [4]. Studies have shown contextual fit can influence effectiveness [5, 6] as well as implementation outcomes such as fidelity [7]. Contextual fit is also a key construct in existing implementation science frameworks, including the Consolidated Framework for Implementation Research [8], the Exploration, Preparation, Implementation, Sustainment Framework (EPIS) [9, 10], and the Replicating Effective Programs (REP) Framework [11, 12]. Growing interest in the role of contextual fit in EBP implementation has increased focus on measuring contextual fit and understanding its influence on implementation outcomes.

A number of measures of contextual fit have been developed and tested in school, mental health, and health care settings. For example, Horner, Salentine, and Albin [13] developed a 16-item Self-Assessment of Contextual Fit (SACF) scale for use in school settings. The SACF assesses fit-related factors such as knowledge of key intervention elements (i.e., behavioral support plans), implementation skills, attitudes and values alignment, resource availability, administrative support, perceived effectiveness, and efficiency. The SACF has been shown to correlate with implementation fidelity in school settings [14], but it has not been tested outside of educational settings. Aarons and colleagues [15] included a contextual fit sub-scale as part of the Evidence-Based Practice Attitude Scale (EBPAS), developed to assess mental health counselors’ attitudes toward EBPs. Within the EBPAS, contextual fit is considered in terms of the values and needs of the client and clinician, including autonomy in EBP selection and how it was used. Recognizing the lack of psychometrically sound and conceptually clear fit measures, Weiner and colleagues [16] developed three different measures of fit for mental health professionals, capturing the concepts of acceptability (personal perceptions of EBP fit), appropriateness (technical and social perceptions of fit), and feasibility (perceptions of practicality), which are often central in pilot research as key indicators of implementation success. Similarly, Kegler and colleagues [17] operationalized CFIR’s intervention fit-related constructs of *complexity* and *compatibility* for use in promoting colorectal cancer screening in federally qualified health centers (FQHCs). Few studies have examined fit measures in HIV prevention and care settings or assessed perceptions of fit in this context.

In this article, we report the results of modifying the SACF for use in HIV clinical settings (hereafter referenced as SACF-HIV); we chose to adapt SACF because of its focus on a youth-centered evidence-based strategy as well as a call for testing it in other contexts [4]. This research was conducted in the context of the Adolescent Medicine Trials Network for HIV/AIDS Interventions (ATN) Scale it Up (SIU) project [18]. An overarching theme of SIU was using implementation science to study the integration of EBPs into HIV clinical settings using the Exploration, Preparation, Implementation, Sustainment (EPIS) Framework [9, 10]. The EPIS Framework [9, 10] highlights four phases in the implementation process and factors within an organization (i.e., the inner context, such as organizational leadership and clinician characteristics) and outside an organization (i.e., outer context, including the service context, policy environment, and EBP consumers), the relationships between individuals and organizations that bridge the inner and outer contexts, and features of the EBP itself (i.e., innovation factors, including the EBP’s fit within the service system and target population). As part of the EPIS project [19], we assessed the factor structure, reliability, and validity of the SACF-HIV for the implementation of tailored motivational interviewing (ATN 146 “TMI”) [20], a set of implementation strategies to train HIV providers in the use of motivational interviewing [21] in adolescent HIV clinical care settings. We also examined variation in perceived fit of TMI and a second evidence-based practice implemented in SIU, The Young Men’s Health Project (ATN 145 “YMHP”), which featured a comparative effectiveness trial examining clinic versus telephone delivery of a four-session YMHP intervention to reduce the risk of HIV infection among young men who have sex with men [22].

## Methods

### Aim, design, setting

The aims of this study were to (1) adapt the SACF for use in HIV clinical settings and examine its psychometric properties and (2) explore perceptions of conceptual fit for two evidence-based interventions. Specific study questions include the following: What is the internal consistency reliability and factor structure of the SACF-HIV? What is the concurrent validity of the SACF-HIV scale? How do perceptions of fit vary, if at all, across two evidenced-based HIV prevention interventions? We hypothesized the scale would be adaptable for HIV-related interventions and settings and that it would be positively related to the EBPAS Fit sub-scale given some conceptual overlap in items regarding dimensions such as fit with client needs and treatment philosophy and other sub-scales assessing fit-related constructs, such as the EBPAS Appeal and Organizational Support sub-scales. Finally, we anticipated a negative or no correlation with sub-scales that assessed constructs in opposite ways (Burden) or those that did not include conceptual overlap, such as EBPAS Balance and Limits sub-scales.

Data for this study stem from ATN 153 [19], a larger cross-project implementation science study within the Scale It Up project using a convergent parallel mixed methods design being implemented in 12 HIV prevention and clinical care settings across the USA, including Baltimore, MD; two sites in Birmingham, AL; New York City, NY; Detroit, MI; Los Angeles, CA; Memphis, TN; Miami, FL; New Orleans, LA; Philadelphia, PA; San Diego, CA; Tampa, FL; and Washington, DC.

### Sample

The study sample was drawn from 12 sites taking part in Scale it Up. All staff and medical providers at the site at the time of baseline were eligible and invited to take part. The sample included clinicians, community health workers, interventionists, adherence counselors, and other members of the prevention and care team who engage in the implementation of evidence-based programs; administrative and research staff with key decision-making roles (e.g., division chief and clinic director) who provide input on prevention and care services and site operations; and a clinical leader (“Site PI”) to represent the organizational leadership perspective.

### Measures

We assessed perceived contextual fit by adapting a version of the SACF in school measure developed by Horner and colleagues [13]. As part of the adaptations, we shifted the focus on behavioral support plans in the original instrument to reflect the specific EBP being assessed (TMI and YMHP); other modifications include changing students to young people or youth and schools to clinics. Several items were dropped that did not transfer well to this study and/or clinic settings (e.g., My school provides the funding, materials, and space needed to implement this behavioral support plan). Participants were given written details (one page) about each evidence-based practice that was planned to be implemented at their clinics (11 clinics for TMI and 3 for YMHP—two sites implemented both EBPs) and asked to use a 5-point Likert scale to rate each EBP separately based on the extent to which they have the skills required to implement the EBPs, their comfort with the different elements of the EBPs, consistency of the EBPs with current clinical practices, ease of implementation including the availability of resources and administrative support for the implementation of the EBPs, and perceived efficacy of the EBPs. Scale ratings ranged from 0 (not at all) to 4 (very great extent) to align with other scales being administered at the same time.

We assessed attitudes toward evidence-based practices using the EBPAS-50 [15]. For this study, we focused on six sub-scales from the EBPAS, including EBP *Fit* with the values and needs of the client and clinician (*α* = .88) [15], the *Balance* of skills and the role of science in treatment (*α* = .79), time and administrative *Burden* associated with learning EBPs (*α* = .77), EBP *Limitations* and their inability to address client needs (*α* = .92), *Appeal *of EBP (*α* = .80), and *Organizational Support* for learning an EBP (*α* = .85). Scale ratings ranged from 0 (not at all) to 4 (very great extent). Cronbach’s alphas reported above reflect those of the scale authors [15]; in the current study, alphas ranged from .63 (*Balance*) to .91 (*Fit*) [23].

### Data collection

At baseline, each participating ATN site provided a list of the identified key stakeholders, including names, contact information (phone number and email), and role(s) within the clinic. These potential participants then received an enrollment email that invited them to join the EPIS study and provided consent information and instructions on joining. Potential participants who did not respond to the initial invitation received reminder emails every 2 weeks throughout the baseline study period to encourage participation. EPIS study enrollment included both qualitative interviews (conducted first) and quantitative surveys (sent following the interviews). We used Qualtrics to collect all survey data, with each participant receiving a unique survey link that included a personal and site identifier. Those not completing the survey within 2 weeks received multiple reminders during the data collection period.

### Analysis

For aim 1 (psychometric properties), we assessed the internal consistency of the SACF-HIV for the TMI intervention only using Cronbach’s alpha and examined the sub-dimensionality with an exploratory factor analysis using principal axis factoring with Promax oblique rotation. Criteria for item inclusion on a factor included primary loadings above 0.40 and cross-loadings below 0.30 [24].

To explore concurrent validity, we examined the correlations between the SACF-HIV (for the overall scale and sub-scales) and six existing sub-scale scores from the EBPAS-50 (*Fit, Balance, Burden, Limits, Appeal, Organizational Support*) for TMI only using Pearson’s correlation coefficient. For aim 2 (variation in perceptions of fit by intervention), we generated descriptive statistics summarizing the SACF-HIV items and the overall scale scores by EBP (TMI and YMHP). All analyses used SPSS statistical software (IBM SPSS Statistics for Windows, version 26).

## Results

### Sample demographics

Participants (*N*=128) were, on average, 40.90 years old (SD = 11.17). Most (81%, *n* = 104) identified as female and Black/African American (41%, *n* = 52) or White (39%, *n* = 49). Most (60%, *n* = 77) had attained advanced educational degrees (i.e., Masters and/or doctorates) and reported an average of 7.04 years (SD = 6.25) experience in their current role and 11.02 years (SD = 7.69) in the field of HIV. Most (81%) were clinical staff (36% were clinical care providers, 30% were psychosocial providers, and 15% were clinical support staff); the remaining participants were administrative staff (18.8%). About a third (33%, *n* = 42) had a caseload exceeding 90 patients, regardless of position, while about 14% reported not having a caseload. The study sample represents 49.6% of those contacted to join the study at baseline (baseline data collection occurred from June 2017 through June 2018).

### Psychometric properties of SACF-HIV TMI

#### Reliability and factor structure

Cronbach’s alpha for the overall SACF-HIV TMI scale was 0.895 (Table 1). The initial exploratory factor analysis for TMI resulted in three factors: one with seven general fit items (skill, experience, comfort, values and client alignment, and overall fit); a second with three implementation support items; and a third factor that had only two items focused on efficiency regarding implementing the EBP. To avoid a two-item sub-scale, the items were forced into a 2-factor solution (Table 1), yielding one *General Fit* factor with nine items (loadings ranging from .57 to .84 for perceptions of skill, experience, comfort, values alignment, client alignment, and overall fit) and a second factor with three items related to *Implementation Support* (loadings ranged from .89 to .97 for resources and administrative support). The variance explained by the final 2-factor solution was 62.44%. Cronbach’s alphas for the sub-scales were 0.877 for *General Fit* and 0.910 *for Implementation Support*.

**Table 1:** Results from an exploratory factor analysis of the Self-Assessment of Contextual Fit-HIV (SACF-HIV) scale for tailored motivational interviewing (TMI)

SACF-HIV TMI items	Factor loading	
	1	2
**Factor 1: General Fit (Cronbach’s alpha = 0.877)**		
1. I believe I have or can gain the skills needed to implement MI.	**.60**	.12
2. I believe I have implemented MI in the past.	**.57**	−.07
3. I believe I would be comfortable implementing the different elements of MI.	**.83**	.05
4. As far as I can tell, the elements of MI are consistent with the way I believe youth should be treated.	**.70**	.10
8. I believe MI is in the best interest of the young people served at this clinic.	**.84**	.02
9. MI is likely to help young people manage their health.	**.75**	.11
10. I think implementing MI would be fairly easy.	**.77**	−.13
11. MI doesn’t seem like it will take a lot of time to implement.	**.66**	−.16
12. Overall, as far as I can tell, I think MI will fit well within our clinic.	**.80**	.08
**Factor 2: Implementation Support (Cronbach’s alpha = 0.910)**		
5. My clinic would ensure staff get trained before implementing MI.	−.01	**.89**
6. My clinic would provide staff time to practice MI before implementing it with clients.	−.03	**.94**
7. My clinic would provide the supervision support needed for effective implementation of MI.	−.06	**.97**

*N* = 115. The extraction method was principal axis factoring with an oblique (Promax with Kaiser Normalization) rotation. Factor loadings above .40 are in bold. Cronbach’s alpha for overall SACF-HIV scale = 0.895. Scale adapted from Horner, Salentine, & Albin, 2003

#### Concurrent validity

Table 2 highlights the results of the correlation analyses examining the relationship between the overall SACF-HIV TMI scores and scores on other selected sub-scales from the EPBAS-50 for TMI. As predicted, the overall SACF-HIV TMI score positively and significantly correlated with TMI EBPAS-50* Fit* (*r* = .33, *p* < 0.001), *Appeal* (*r* = .35, *p* < 0.001), and *Organizational Support* (*r* = .35, *p* < 0.001) and negatively and significantly correlated with EBPAS-50* Burden* (*r* = −.21, *p* < 0.05). It was not significantly correlated with EBPAS-50* Balance* (*r* = .17) *or Limitations* (*r* = −.11).

**Table 2:** EBPAS-50 sub-scale means and Pearson’s correlation coefficients between EBPAS-50 sub-scales and SACF-HIV sub-scales for TMI: baseline (*N*=115)

Variable	Mean	SD	Correlation: overall SACF-HIV score	Correlation: SACF-HIV General Fit sub-scale	Correlation: SACF-HIV Implementation Support sub-scale
EBPAS Fit	3.31	0.69	.33***	.38***	.11
EBPAS Balance	2.31	0.68	.17	.17	.11
EBPAS Burden	0.67	0.74	−.21*	−.20*	−.15
EBPAS Limits	0.51	0.88	−.11	−.16	.05
EBPAS Appeal	3.24	0.64	.35***	.37***	.20*
EBPAS Organizational Support	2.57	0.96	.35***	.38***	.16

Significance of correlations: ^*^p < .05. ^**^p < .01. *** *p* < .001

Correlation analyses with the SACF-HIV TMI G*eneral Fit *sub-scale followed a similar pattern to the overall SACF-HIV TMI (Table 2). The SACF-HIV *Implementation Support* sub-scale was not significantly correlated with EBPAS-50 *Fit*, *Balance*, *Burden*, *Limits*, or *Organizational Support*, but was correlated with *Appeal* (*r* = .25, *p* < 0.05).

### Adapted SACF scores by evidence-based intervention

Table 3 shows the adapted SACF-HIV item frequencies, mean scores, and standard deviations for TMI and YMHP separately. Item mean scores for SACF-HIV TMI ranged from 2.03 (SD=1.14) to 3.12 (SD=0.97); mean scores and standard deviations for SACF-HIV YMHP ranged from 1.88 (SD=1.19) to 2.96 (SD=1.00). As noted in Table 4, the overall mean fit scores for TMI and YMPH are 2.78 and 2.53, respectively, and this difference was statistically significant (*p* < 0.05).

**Table 3:** Item frequencies and mean scores for Self-Assessment of Conceptual Fit-HIV (SACF-HIV) scale items: baseline (TMI *N*=115; YMHP *N*=24)

Items	Strategy	Not at all (0)	Slight extent [1]	Moderate extent [2]	Great extent [3]	Very great extent [4]	Item average (SD)
I believe I have or can gain the skills needed to implement … .	MI	0.9%	7.8%	12.2%	36.5%	42.6%	3.12 (0.97)
	YMHP	0%	4.2%	37.5%	37.5%	20.8%	2.75 (0.85)
I believe I have implemented … in the past.^a^	MI	13.9%	20.9%	17.4%	24.3%	23.5%	2.23 (1.38)
	YMHP	20.8%	20.8%	29.2%	12.5%	16.7%	1.83 (1.37)
I believe I would be comfortable implementing the different elements of … .^a^	MI	0%	7.0%	19.1%	32.2%	41.7%	3.09 (0.94)
	YMHP	0%	12.5%	41.7%	20.8%	25.0%	2.58 (1.02)
As far as I can tell, the elements of … are consistent with the way I believe youth should be treated.	MI	0%	5.2%	20.9%	34.8%	39.1%	3.08 (0.90)
	YMHP	0%	12.5%	29.2%	29.2%	29.2%	2.75 (1.03)
My clinic would ensure staff get trained before implementing … .	MI	0.9%	7.8%	20.9%	33.9%	36.5%	2.97 (0.99)
	YMHP	4.2%	0%	25.0%	37.5%	33.3%	2.96 (1.00)
My clinic would provide staff time to practice … before implementing it with clients.	MI	0.9%	11.3%	23.5%	38.3%	26.1%	2.77 (0.99)
	YMHP	0%	0%	29.2%	45.8%	25.0%	2.96 (0.75)
My clinic would provide the supervision support needed for effective implementation of … .	MI	2.6%	12.2%	26.1%	33.9%	25.2%	2.67 (1.07)
	YMHP	0%	0%	33.3%	41.7%	25.0%	2.92 (0.78)
I believe … is in the best interest of the young people served at this clinic.	MI	0%	5.2%	21.7%	32.2%	40.9%	3.09 (0.91)
	YMHP	0%	4.2%	54.2%	29.2%	12.5%	2.50 (0.78)
… is likely to help young people manage their health.	MI	0%	4.3%	23.5%	33.0%	39.1%	3.07 (0.90)
	YMHP	0%	4.2%	41.7%	37.5%	16.7%	2.67 (0.82)
I think implementing … would be fairly easy.	MI	2.6%	14.8%	41.7%	26.1%	14.8%	2.36 (0.99)
	YMHP	8.3%	12.5%	45.8%	25.0%	8.3%	2.13 (1.04)
… doesn’t seem like it will take a lot of time to implement.	MI	8.7%	26.1%	28.7%	26.1%	10.4%	2.03 (1.14)
	YMHP	16.7%	20.8%	25.0%	33.3%	4.2%	1.88 (1.19)
Overall, as far as I can tell, I think … will fit well within our clinic.	MI	0%	5.2%	25.2%	40.0%	29.6%	2.94 (0.87)
	YMHP	0%	12.5%	41.7%	33.3%	12.5%	2.46 (0.88)

Scale range is not at all (score of 0) to very great extent (score of 4)

^a^Item level mean comparison differences significant at *p* < 0.05 via Wilcoxon signed rank test (*n*=18)

**Table 4:** Self-Assessment of Contextual Fit HIV (SACF-HIV) scale means overall and by site: baseline

Sites	Mean score TMI (*N*=115)	Mean score YMHP (*N*=24)
Overall^a^	**2.78 (0.691)**	**2.53 (0.650)**
Site 1	2.78 (0.885)	NA
Site 2	2.96 (0.443)	NA
Site 3	3.07 (0.649)	NA
Site 4	NA	2.65 (0.684)
Site 5	2.95 (0.555)	NA
Site 6	3.09 (0.410)	NA
Site 7	2.60 (0.382)	2.52 (0.450)
Site 8	2.28 (1.219)	NA
Site 9	2.77 (0.635)	2.48 (0.733)
Site 10	2.92 (0.646)	NA
Site 11	2.24 (0.843)	NA
Site 12	2.66 (0.653)	NA

Mean score based on a scale range of 0=not at all, 1=slight extent, 2=moderate extent, 3=great extent, and 4=very great extent

^a^Overall mean comparison differences significant at *p* < 0.05 via Wilcoxon signed rank test (*n*=18)

## Discussion

The primary objectives of the current study were to examine the psychometric properties of the SACF-HIV, an adapted Self-Assessment of Contextual Fit measure for HIV clinical care settings, and explore variations in perceptions of contextual fit across two different types of interventions (a structural intervention to train care providers in MI and an individually focused HIV prevention intervention). The internal consistency of the overall scale and the two sub-scales were well within the range considered acceptable [25]. One sub-scale captures general insights regarding contextual fit, such as perceptions of skill, experience, alignment with values and client needs, and efficiency, and the second sub-scale captures perceptions related to implementation support.

Concurrent validity was supported by significant correlations in the expected direction with well-established fit or fit-related sub-scales as measured by the EBPAS-50 and no relationships with others. As an example, the overall score and the *General Fit* sub-score of the SACF-HIV TMI correlated positively with the EBPAS-50 *Fit* score; both sub-scales share conceptual overlap regarding perceptions of fit with client needs, clinical approaches, and treatment philosophy. The EBPAS-50 *Fit* sub-scale assesses other dimensions of fit, such as autonomy in using an EBP and perceptions of clients’ acceptance, which is reflected in the moderate correlation between the two measures. We found a small negative association with the EBPAS-50 *Burden* sub-scale; both scales assess burden, but in slightly different ways and using opposite valances (EBPAS *Burden* focused on negative burdens, whereas the SACF-HIV focused on ease of implementation). Similarly, we hypothesized that there would be little relationship between SACF-HIV and the EBPAS-50 *Limits* and *Balance* sub-scales for TMI given the lack of conceptual overlap between the measures, and this was confirmed. One may have anticipated a stronger positive correlation between the SACF-HIV *Implementation Support* sub-scale and the *Organizational Support* sub-scale on the EBPAS; however, the focus of the items varied somewhat—the SACF-HIV *Implementation Support* items centered on perceived clinic support whereas the EBPAS-50 *Organizational Support* items focused on individuals’ desire to learn the evidence-based intervention given organizational supports.

One of the challenges of assessing contextual fit is the lack of accord and research on core elements of contextual fit. Horner, a lead developer of the SACF, and colleagues [4] identify eight elements important in establishing contextual fit between an intervention and setting: need, precision, an evidence-base, efficiency, skills/competencies, cultural relevance, resources, and administrative and organizational support. While there are no current contextual fit measures that capture all eight of these elements, the SACF-HIV captures many using an abbreviated approach, underscoring its promise as a measure of contextual fit.

Some of these elements, such as precision, were provided as part of data collection (in the description of TMI and YMHP), but we did not assess perceptions of the extent to which implementers felt the core features of the interventions were well defined and this would vary based on the degree of familiarity individuals had with the interventions. Assessing perceptions regarding clarity of core components could provide guidance on further intervention refinement, training, and support. Furthermore, the assessment of cultural relevance is relatively limited and focuses on values alignment around how youth should be treated and what might be in their best interest; measurement of this element could delve into assessing other aspects of cultural relevance, such as social and emotional relevance, the extent to which the interventions are affirming of all young people, and the extent to which the interventions accommodate differing cultures and learning differences. Data on these aspects of cultural fit could be beneficial for intervention selection or refinement (e.g., the exploration phase in a model like EPIS) and might also guide training and support during a preparation phase to ensure implementers are able to describe and promote critical intervention features to recruit and engage young people. The SACF-HIV we examined also does not assess perceptions of the evidence supporting the intervention—rather, this information was provided as part of the description of the intervention strategy. Measuring perceptions of an intervention’s underlying evidence could inform users at the exploration and adoption phases of implementation as well as the implementation phase (e.g., users who are less convinced of the evidence may be more inclined to adapt it).

We found that the SACF-HIV captures variations in contextual fit by EBP strategy, which may be an important consideration for shaping training and support plans to scaffold EBP uptake and implementation. As an example, participants rated perceived comfort with delivering key components of TMI more highly than YHMP, and perceived comfort ratings aligned with perceived ratings of skills to implement the two interventions. Motivational interviewing (MI) may be more familiar to stakeholders given it is a client-centered approach that can be incorporated into clinical settings in a variety of ways [26]. It also is perceived to be a good fit with adolescent patient populations given its emphasis on autonomy and self-efficacy [27]. YMHP, on the other hand, is a multi-session program designed for a particular population (young men who have sex with young men). As a result, it may not be as familiar across clinic settings. It may also be perceived to require additional time to implement program sessions versus a strategy like MI, which can be integrated into daily routines. Despite the variation, the range of response was relatively limited, which may have resulted from the fact that most participating sites represented academic partnerships who may be more open to innovation due to their university affiliation.

Assessing contextual fit has relevance across phases of intervention development and implementation. During intervention development, data on fit (e.g., collected during pilot testing) could lead to refinements that boost proximal implementation outcomes such as acceptability [28, 16]. At the exploration phase, data on perceptions of fit could inform decisions regarding EBP adoption. Indeed, in the qualitative interviews stemming from the larger EPIS study (ATN 153), providers underscored the importance of taking the time to share the evidence base when introducing a new EBP and developing clinically relevant examples that align with the specific settings and characteristic patients, such as patient testimonials or brief videos demonstrating EBP integration, to increase EBP acceptability and perceptions of fit [27].

Other tools support the notion of examining fit early, such as the Hexagon Discussion and Analysis tool [29]. Once a program is adopted, fit data could be used to shape implementation resources and supports, which was the focus of the study by Monzalve and Horner [7] who used a contextual fit enhancement protocol to boost the implementation of behavioral support plans, yielding increases in implementation fidelity and student outcomes. Collecting fit data over time might also inform needed EBP refinements and ongoing support for sustainment [4]. The SACF-HIV could be used over time to guide implementation supports and inform intervention refinements in these ways.

### Limitations

The study sample was relatively small, from an intervention study and restricted to key stakeholders from 12 clinics across the USA that generally represent academic partnerships who are likely more open to innovation due to their university affiliation. Insights regarding variation in perceptions of fit by intervention type are limited by the small sub-sample of clinics (three) that were selected to implement YMHP. Because of the small sample, we were unable to explore how, if at all, the factor structure of the instrument differed for that intervention compared to TMI. This study was restricted to baseline assessments only and we did not examine the association of fit with implementation or programmatic outcomes, an important focus for future studies. Nevertheless, the study contributes important data and insights regarding an adapted tool for assessing perceptions of contextual fit for use in HIV prevention and clinical care settings that serve adolescent populations.

### Directions for future research

This study examined baseline perceptions of contextual fit in selected HIV prevention and treatment settings. Additional research could explore how, if at all, perceptions of fit change over time and what factors contribute to such changes. Further research is also needed on how changes in conceptual fit perceptions impact key EBP implementation or effectiveness outcomes, and what strategies might be effective in boosting fit (e.g., adaptations to intervention, training to clarify evidence base and provide more experience with strategies). Finally, future research could also examine the generalizability of these findings in other HIV prevention and clinical care settings, such as those who serve adult populations.

There is also a continuing need for research that examines and clarifies the multiple domains of contextual fit to guide implementation supports and policy in HIV clinical settings. This research would support further refinement and/or development of contextual fit measures.

## Conclusions

Contextual fit is an important variable in the implementation of evidence-based programs (EBPs), yet there are relatively few tools assessing perceptions of fit in HIV clinical settings. This study contributes to the literature by assessing an adapted measure of contextual fit originally developed by Horner and colleagues [13] for school settings and demonstrating its potential use in HIV clinical settings with different evidence-based strategies. The SACF-HIV is a 12-item scale that captures global perceptions of contextual fit and implementation support. The adapted scale can be used by practitioners and/or researchers interested in understanding the context for implementation and/or planning to prepare and support sites for successful implementation.

## Data Availability

The survey is available from the study authors; data from SIU will be available following study end (November 2022).

## Abbreviations

EPIS: Exploration, Preparation, Implementation, Sustainment (Framework)
EBP: Evidence-based program
EBPAS: Evidence-Based Practice Attitude Scale
SACF: Self-Assessment of Contextual Fit
TMI: Tailored motivational interviewing
YMHP: Young Men’s Health Project
